# Epidemiology of Asthma in Children and Adults

**DOI:** 10.3389/fped.2019.00246

**Published:** 2019-06-18

**Authors:** Shyamali C. Dharmage, Jennifer L. Perret, Adnan Custovic

**Affiliations:** ^1^Allergy and Lung Health Unit, School of Population and Global Health, University of Melbourne, Melbourne, VIC, Australia; ^2^Institute for Breathing and Sleep, Melbourne, VIC, Australia; ^3^Department of Paediatrics, Imperial College London, London, United Kingdom

**Keywords:** asthma epidemiology, incidence, prevalence, risk factors, lifecourse

## Abstract

Asthma is a globally significant non-communicable disease with major public health consequences for both children and adults, including high morbidity, and mortality in severe cases. We have summarized the evidence on asthma trends, environmental determinants, and long-term impacts while comparing these epidemiological features across childhood asthma and adult asthma. While asthma incidence and prevalence are higher in children, morbidity, and mortality are higher in adults. Childhood asthma is more common in boys while adult asthma is more common in women, and the reversal of this sex difference in prevalence occurs around puberty suggesting sex hormones may play a role in the etiology of asthma. The global epidemic of asthma that has been observed in both children and adults is still continuing, especially in low to middle income countries, although it has subsided in some developed countries. As a heterogeneous disease, distinct asthma phenotypes, and endotypes need to be adequately characterized to develop more accurate and meaningful definitions for use in research and clinical settings. This may be facilitated by new clustering techniques such as latent class analysis, and computational phenotyping methods are being developed to retrieve information from electronic health records using natural language processing (NLP) algorithms to assist in the early diagnosis of asthma. While some important environmental determinants that trigger asthma are well-established, more work is needed to define the role of environmental exposures in the development of asthma in both children and adults. There is increasing evidence that investigation into possible gene-by-environment and environment-by-environment interactions may help to better uncover the determinants of asthma. Therefore, there is an urgent need to further investigate the interrelationship between environmental and genetic determinants to identify high risk groups and key modifiable exposures. For children, asthma may impair airway development and reduce maximally attained lung function, and these lung function deficits may persist into adulthood without additional progressive loss. Adult asthma may accelerate lung function decline and increase the risk of fixed airflow obstruction, with the effect of early onset asthma being greater than late onset asthma. Therefore, in managing asthma, our focus going forward should be firmly on improving not only short-term symptoms, but also the long-term respiratory and other health outcomes.

## Key Points

- Asthma is a major non-communicable disease affecting both children and adults, with high morbidity and relatively low mortality compared with other chronic diseases.
- The global epidemic of asthma that has been observed in both children and adults is still continuing especially in low to middle income countries, although some evidence suggests it has subsided in some high-income countries.
- Asthma is a heterogeneous disease and distinct asthma phenotypes and endotypes need to be adequately characterized. This may be facilitated by cluster and latent class analysis if clusters/classes are associated with clinically important asthma outcomes.
- Computational phenotyping methods to retrieve information from electronic health records using natural language processing (NLP) algorithms are innovative and may assist in the early diagnosis of asthma and in epidemiological research
- While some environmental triggers are well-established, investigation into possible gene-by-environment and environment-by-environment interactions may help to better uncover the determinants of asthma.
- Work-related asthma from occupational sensitizers (asthmagens) and/or irritants is common and is an important consideration for individuals who present with asthma symptoms during their productive working years
- For children, asthma may impair airway development and reduce maximally attained lung function, and these lung function deficits may track (or persist) into adulthood without additional progressive loss.
- Adult asthma may accelerate lung function decline and increase the risk of fixed airflow obstruction, especially for smokers with asthma
- People with asthma are more susceptible to infections and non-communicable chronic co-morbidities which are associated with worse asthma outcomes
- Defining asthma remains an ongoing challenge and innovative methods are needed to identify, diagnose, and accurately classify asthma at an early stage to most effectively implement optimal management and reduce the health burden attributable to asthma

## Introduction

Asthma is one of the most common major non-communicable diseases and for many, has a substantial impact on quality of life. Globally, asthma is ranked 16th among the leading causes of years lived with disability and 28th among the leading causes of burden of disease, as measured by disability-adjusted life years. Around 300 million people have asthma worldwide, and it is likely that by 2025 a further 100 million may be affected (1). There is a large geographical variation in asthma prevalence, severity, and mortality. While asthma prevalence is higher in high income countries, most asthma-related mortality occurs in low-middle income countries (2). Despite the advances in asthma treatment in recent decades, there are still gains to be made in terms of improving patient education, employing new diagnostic approaches, and implementing personalized case management.

Patterns in asthma incidence and prevalence differ between children and adults. It is well-known that asthma often begins in childhood but can occur at any time throughout life, with some developing asthma for the first time as adults. While asthma incidence and prevalence are higher in children, asthma-related healthcare use, and mortality are higher in adults. Interestingly, incidence and prevalence of asthma differs by sex across the lifespan. Pre-pubertal boys have a higher asthma incidence, prevalence, and hospitalization rate than girls of the same age, but this trend reverses during adolescence (3). Females continue to have a higher burden of asthma than males well into the 5th decade of life. However, the female-male gap in asthma burden narrows around the 5th decade. Some even suggest that the sex differential in asthma incidence may reverse again, following a sharp increase in asthma incidence in males around the 4th decade of life (3). The sex reversal in asthma burden around major reproductive events suggests that sex hormones may play a role in the etiology of asthma.

The current evidence suggests that asthma is a complex multifactorial disorder and its etiology is increasingly attributed to interactions between genetic susceptibility, host factors, and environmental exposures. These include environmental factors (air pollution, pollens, mold and other aeroallergens, and weather), host factors (obesity, nutritional factors, infections, allergic sensitization), and genetic factors (asthma susceptibility loci on genes). Although underlying mechanisms of asthma are not yet fully understood, they may include airway inflammation, control of airway tone and reactivity (4). It is also now recognized that asthma may not be a single disease but a group of heterogeneous phenotypes with different etiologies and prognoses (5). While phenotyping individuals with asthma has been used to help guide clinical management, defining the entity of “asthma” has been a major challenge encountered in research, especially in epidemiological research, where in-depth data collection needs to be balanced with the large number of study participants necessary for adequate power.

This is not an exhaustive or systematic review on all the complexities of asthma epidemiology but aims to provide an epidemiological perspective by comparing and contrasting trends, and discussing the current debate on definitions, environmental risk factors, and long-term consequences of childhood and adult asthma. The roles of genetic factors and gene-environment interactions in the etiology of asthma are described in another article in this series and are therefore not addressed here. Similarly, an article published alongside this article will be covering asthma categories, phenotypes and endotypes, although these topics have been introduced in the present review.

## Global Epidemic of Asthma Prevalence—Subsiding in Some Parts of the World

During the second half of the Twentieth century, notably since the 1960s, a sharp increase in asthma prevalence was observed in a number of developed countries. This observation was a result of repeated cross-sectional surveys of prevalence of asthma, mainly in children but also in adults. As a result of this observation, in the 1990s, a series of epidemiological studies were established across the world to estimate global asthma prevalence and incidence, and identify risk factors associated with these outcomes. These include large multinational studies in children [such as the International Study of Asthma and Allergies in Childhood (ISAAC; http://isaac.auckland.ac.nz/) (6–8)] and in adults [such as the European Community Respiratory Health Survey (ECRHS; http://www.ecrhs.org/) (9)]. These studies confirmed that asthma is one of the most common chronic diseases across the globe in all age groups and there is substantial variation in asthma prevalence worldwide. It is now acknowledged that the prevalence of both childhood and adult asthma may have peaked in some areas, predominantly in high-income countries, whereas an increase may be continuing in low and mid-income countries (10). It is important to note that a reduction in the prevalence of current asthma is determined by improved asthma control and/or reduced asthma incidence at a population level. Thus, a reduction in prevalence of current asthma may well-reflect improved asthma control through increased medication use from more widespread prescribing habits and better compliance. Documenting reductions in asthma incidence is complicated as parallel cohort studies with specific age windows are needed to establish patterns with the comparison group ideally from the same geographical region. These challenges might in part explain why studies from Australia and UK have not consistently shown reductions in asthma prevalence and why temporal trends in European and Asian countries between the 1970s and mid-2000s have been conflicting (4).

Although greater awareness, recognition, and/or diagnostic shifts have been suggested as contributory factors to the steep rise in asthma prevalence observed over the last four decades of the Twentieth century, repeated cross-sectional surveys using objective measures, such as bronchial hyperreactivity, have confirmed that these factors are unlikely to fully explain this epidemic (4). Though the specific elements driving this rise in prevalence have not been established, it is now clear that the reasons almost certainly are linked to changing environmental factors, acting through gene-by-environmental interactions. Given the rapidity with which the prevalence has risen, this argues against alterations to the population's genetic makeup alone.

The increase in asthma prevalence has been paralleled by a similar increase in other allergies such allergic rhinitis and eczema (11). Multiple hypotheses have been proposed to explain this epidemic, and these have been investigated but are still debated in the field. In the late 1980s, it was thought that increased exposure to indoor allergens such as house dust mite, cat, and fungi due to modernization of housing with tighter insulation and the use of plush furniture and carpets may have contributed to increases in asthma and allergies. Also, in 1989, Strachan proposed the “hygiene hypothesis,” suggesting that decreased exposure to unhygienic environments in early life may have led to the increased prevalence of these conditions (12). In 2003, Rook et al. proposed a lack of exposure to non-pathogenic microbes and commensal organisms as an alternative explanation for the increased prevalence of asthma and allergic diseases (13). This led to the “microbial diversity” hypothesis that suggests that environments rich in microbial diversity in the gut mucosa and respiratory tract are the key factors in priming and regulating the immune system.

Asthma mortality and hospitalization rates with acute severe asthma attacks also increased in all age groups during the period from 1960 to 1985, with the highest rates of increase in young pre-school children (14). Following this period, during the 1990s and early 2000s, a decreasing trend in severity has been observed. However, despite novel treatments and improved inhalers for the administration of topical therapies, no further improvements in either mortality or hospitalization rates have been observed in the last decade, either in children or in adults (15).

Given that some childhood asthma persists into adulthood, it is possible that the “asthma epidemic” in children during the 1980–90s has subsequently translated into an increased adult prevalence. However, establishing this trend is challenging due to increased trends also affecting adult asthma, variable asthma definitions, heterogeneity of asthma phenotypes, and limited sequential studies within distinct geographical regions.

## Epidemiological Definitions of Asthma—Part of the Challenge

Definitions are key to our understanding of the epidemiology, pathophysiology and etiology of asthma, and ascertaining similarities or differences between childhood and adult asthma. Yet variation in asthma severity, age-of-asthma onset, allergic vs. non-allergic phenotypes and type of airway inflammation add complexity to the standard definitions used in large population-based studies (16).

Despite attempts to reach a consensus definition for epidemiological studies, as many as 60 different definitions of “childhood asthma” have been used across 122 published studies (17). Although some of these definitions may appear almost identical, the multiplicity in the way the primary outcome is defined can have a substantial impact on the estimated prevalence and risk factors. As an example, the above study has shown that the agreement between four seemingly very similar and commonly used definitions was overall relatively low (61%), and well-over a third of children in a study could move from being considered “asthma cases” to “controls” depending on the definition used (17). These differences need to be considered when interpreting results of meta-analyses of asthma epidemiology.

Some epidemiological definitions are more sensitive while others are more specific, with both scenarios leading to misclassification of asthma status. For example, current asthma defined by “wheezy breathing in the last 12 months in the absence of a cold” is a more sensitive definition than using “doctor-diagnosed asthma” as they do not rely on the individual to seek health advice (18), while the latter is a more specific definition. As such, survey definitions that adopt wheezy breathing effectively estimate a greater asthma prevalence than clinical definitions which may also incorporate objective measures such as the co-presence of bronchial hyperreactivity (19).

Furthermore, it is important to consider the age of the participants. Particularly for early childhood cohort studies, it can be difficult to distinguish between transient wheeze precipitated by viral infections and the onset of true asthma in young children, although in many cases, recurrent wheezing episodes during the first few years of life can represent the early stages of asthma. For adults, prospectively collected data on childhood asthma status can minimize the risk of recall bias, otherwise retrospective recall typically misclassifies relapsed childhood asthma as late-onset asthma and preferentially favors those who have more severe childhood disease (20). For older people at risk of co-morbidity, an asthma diagnosis may be difficult to differentiate from other diseases causing breathlessness, especially chronic obstructive pulmonary disease (COPD), and heart failure.

A consolidated definition of asthma may not be desirable given the emerging consensus in the research community that “asthma” is an umbrella term for several diseases with similar clinical manifestations but different underlying pathophysiological mechanisms (5), often referred to as “asthma endotypes” (21, 22). In this context, symptoms associated with asthma (such as wheeze or cough) and objective measures (such as lung function and biomarkers in blood, exhaled breath, sputum, and/or urine) should be viewed as observable traits (or “phenotypes”) (23, 24). However, it is important to note that different mechanisms may give rise to similar or almost identical observable traits, while the same underlying mechanism may also result in distinct phenotypes in different patients (25).

To date, the framework of asthma endotypes remains a theoretical concept (23), but this framework may also help in developing accurate asthma definitions to facilitate further discovery of their underlying mechanisms (23). With increasing interest in endotypes, there have even been calls to abolish the term “asthma” altogether. However, the term “asthma” provides a practical and functional framework for clinicians to manage patients and for scientists to search for mechanisms; and before abolishing it, we first need to propose more useful and meaningful terminology, which will only come through a more thorough understanding of asthma endotypes.

To further this concept, asthma heterogeneity that features multiple different subtypes has major implications for future studies. However, phenotyping asthma from questionnaire data alone seems increasingly insufficient. While previous cluster analyses have been used to identify patient clusters based on asthma symptoms and airway eosinophilia (16), newer statistical techniques such as latent class analyses (LCA) also have the potential to effectively deal with asthma heterogeneity. Essentially, LCA methods are able to identify novel and statistically distinct classes among individuals in a relatively unbiased way, and are based on measured variables that relate to asthma symptoms (26) and/or biomarkers such as bronchial hyperresponsiveness and atopy (27, 28). A notable example that extended the knowledge of the observed wheezing phenotypes in childhood from the TAHS cohort (29) identified a new phenotype known as “intermediate onset wheezers” (30). This class was subsequently found to have persistent deficits in post-bronchodilator FEV_1_ in adolescence (31). Thus, while LCA can readily document asthma heterogeneity, it is of most value if associations are shown between the LCA classifications and clinically important asthma outcomes. To assist in the early identification and diagnosis of asthma, there are currently available innovative computational phenotyping methods that leverage complex electronic health record data that have been validated in different practice settings (32, 33). Using natural language processing (NLP) algorithms, asthma is identified via automated chart review based on predetermined asthma criteria (PAC) via a two-step process: (1) finding asthma-related concepts in text that match specified criteria, then (2) assigning an asthma status classification to individual records (34). While this artificial intelligence algorithm is being developed to improve overall asthma care as a population management tool, it can potentially retrieve information for large-scale, multi-center population studies which has previously been an underutilized data source for asthma research.

## Severe Asthma in Adults and Children

Severe asthma represents a small subgroup of individuals who have a disproportionately high health burden. The European Respiratory Society (ERS)/American Thoracic Society (ATS) Task Force defines severe asthma as “asthma which requires treatment with high dose of inhaled corticosteroids (ICS) plus a second controller (and/or systemic corticosteroids) to prevent it from becoming “uncontrolled,” or which remains “uncontrolled” despite this therapy” (35). This definition applies to both children and adults with asthma, and it is highly likely that the condition we refer to as “severe asthma” is the extreme end of the spectrum for several different asthma endotypes.

There is considerable variation in the prevalence estimates of severe asthma. For example, in has been reported that 4.2% of Swedish adult asthmatic patients in primary care settings have severe disease (36). Surveys in Denmark described a higher proportion of ~8% of severe asthmatics (37), while some studies report that as high as 20% or even more than 30% of asthmatic patients have at least some features of severe disease (38, 39). The proportion of severe asthmatics appears lower in childhood asthma compared to adult asthma (40). For example, in a birth cohort in Sweden, only seven of 329 12-year old asthmatic children had severe asthma as defined by the World Health Organization (WHO) (41), suggesting a prevalence of 0.23% in the general population and 2.1% among children with asthma (42). Among 616 children in a Norwegian birth cohort, 67 had asthma, of whom only three were defined as having a severe disease, with an estimated population prevalence of severe asthma at age 10 years of 0.5, and 4.5% among asthmatic children (43). A study conducted within a birth cohort in Manchester (UK), identified a latent class of persistent troublesome wheezers, comprising children with high number of acute asthma attacks, hospital admissions, and unscheduled healthcare visits, which accounted for ~10% of children with doctor-diagnosed asthma, and 3.2% of the general population (44). These children exhibited numerous features associated with severe asthma including diminished lung function, high FeNO and hyperreactive airways (44), with a significant loss of lung function between preschool and mid-school age (45). However, when “severe asthma” was defined using ERS/ATS (35) or WHO (41) criteria, only a small number of children were classified as having severe asthma, suggesting that we need to look beyond the amount of medication and disease control when defining severe disease (46).

In the absence of linking data with national pharmaceutical schemes, capturing detailed information on medication use is challenging in epidemiological studies, although such information is critical when defining severe asthma. For example, in children the “maximum treatment” used to define severe asthma includes high doses of ICS or oral corticosteroids, often in combination with add-on therapy with long-acting β-2 agonists (LABA) and/or leukotriene-receptor antagonists (LTRA) (47–49). The limitation of the use of “maximum treatment” is that there may be different reasons for poor asthma control among patients on “maximum treatment,” such as the wrong diagnosis (46), non-adherence with medication (50), or therapy-resistant disease (48).

To provide a more useful clinical and research framework for the investigation of severe childhood asthma, Bush et al. have proposed the term “Problematic severe asthma” (PSA) for children who require specialist referral because of the apparent poor response to maximum asthma treatment (48, 49). Once other potential causes of asthma-like symptoms are excluded and asthma diagnosis is confirmed, children with PSA can be broadly divided into three distinct (but occasionally overlapping) groups: Difficult-to-treat (or difficult) asthma (DA); Asthma with co-morbidities (“Asthma plus”); and Severe therapy-resistant asthma (STRA) (49). The main characteristics of DA are that principal factors which contribute to troublesome symptoms are potentially modifiable. These include poor adherence with medication (51), ongoing exposure to adverse environmental factors such as allergens (52–54), tobacco smoke (55), and air pollution (56–58), and psychosocial factors (59–61). If these modifiable factors are addressed, this should result in better asthma control, including improvement in symptoms, and reduction in severe asthma attacks (62, 63). Children with troublesome asthma and comorbid conditions such as food allergy (64), allergic rhinitis (65), and/or obesity (66) are considered to have “Asthma plus” (i.e., asthma + comorbidities). Treatment of these disorders co-occurring with asthma may improve asthma control (65), although there is a paucity of evidence to support this from well-designed randomized intervention trials (67). However, despite interventions and treatments aimed at addressing modifiable factors and comorbidities, some children with DA and Asthma plus may not improve [e.g., because of the continued poor adherence with medications (50, 68), or ongoing high exposure to allergens (69–71)], in which case they should be considered as having refractory DA or refractory Asthma plus (46, 72).

Although there is considerable within-group heterogeneity in each of the above categories, and strict differentiation may be challenging and on occasion not possible, the concepts which distinguish PSA, DA, and STRA are useful in both a research and clinical context (73), and can also be used in adult severe asthma (74).

A number of studies have described differences between childhood and adult severe asthma based on symptom patterns. Severe asthma is predominantly persistent in adults, but much more variable with rapidly evolving severe attacks in children, often remaining symptom-free between the attacks (75, 76). However, we would argue that these differences may have been over-emphasized. It is possible that severe asthmatics who are currently seen in adult clinics reflect the patterns seen in pediatric clinics 10–20 years ago, and that the pattern of severe disease currently seen in pediatric severe asthma clinics may be foreshadowing the severe adult asthma in years to come. It is possible that the differences observed in cross-sectional studies carried out contemporaneously in children and adults can in part be explained by a cohort effect.

Impaired innate anti-viral immunity with diminished interferon induction to rhinovirus has been reported in both children and adults with severe asthma (77–79). A recent study has identified different patterns of cytokine responses by blood mononuclear cells after stimulation with rhinovirus-16 between children with early-onset troublesome asthma compared to those with late-onset mild allergic asthma (80). The synergism between allergic sensitization, high allergen exposure, and viral infection (mostly rhinovirus) has been shown to increase the risk of hospitalization, both in children (52) and in adults (81) with asthma.

One factor strongly associated with severe asthma in children and adolescents is allergic sensitization (82–87). Several studies in recent years have suggested that there may be different classes of sensitization and that some of these sensitization subtypes are more pathologic than others (85, 88, 89). Further studies using component-resolved diagnostics rather than standard skin and blood tests to whole allergen extracts have identified different cross-sectional and longitudinal patterns of component-specific IgE responses associated with different risk of asthma presence, persistence and severity in children (90–93). If the above notion is correct, this may be an indicator of adult severe asthma research and practice in years to come.

## Environmental Exposures Associated With Asthma in Children and Adults

Childhood asthma and adult onset asthma are known to share many of the same causes and triggers. While there is stronger evidence on the role of environmental factors as triggers than causes, there is increasing evidence for interactions among and between environmental and other intrinsic factors, such as genetics and atopy, to potentially cause asthma. The vast majority of childhood onset asthma manifests as an allergic phenotype, while there is a predominance of the non-allergic phenotype in adult onset asthma. However, both allergic and non-allergic asthma can exhibit individual responses to both allergic and non-allergic airborne triggers such as animal hair and dander, pollen, and mold (fungal) spores, food allergens, tobacco smoke, or other pollutant exposures (Table 1). Other than this table that provides key references to the main environmental exposures associated with asthma across the lifespan, the typical non-allergic, food and animal triggers of asthma are not described further in this chapter. Subsequent text has focused on the relationships between outdoor, indoor and workplace air pollutants and allergens and asthma, followed by a section on lifestyle factors such as obesity, diet, and breastfeeding.

**Table 1:** Environmental exposures associated with asthma spanning childhood to adulthood.

**Environmental exposure**	**Feature**	**Children**	**Adolescents**	**Adults**
**ALLERGIC**				
Airborne triggers - House dust mite (HDM) - Animal hair and dander - Pollen exposure - Mold (fungal) spores - Thunderstorm asthma	HDM aeroallergen is a perennial asthma trigger linked to asthma incidence in high-risk children	(94–96)	(94)	(94)
	Association between pet allergen exposure and asthma is conflicting and non-conclusive	(94, 97, 98)	(98)	(98)
	Grass pollen triggers asthma exacerbations requiring emergency department attendances	(99)	(99)	
	Indoor fungal spore exposure can worsen asthma control; decreased visible indoor mold reduces symptoms but not PEFM variability	(100, 101)	(100, 101)	(100, 101)
	High outdoor Alternaria exposure may contribute to severe asthma/ respiratory arrest (ages 11–25)	(102)	(103)	(103)
	Fungal spores, especially Cladosporium are associated with asthma hospitalization (ages 2–17)	(104)	(104)	
	IgE sensitization to mycoses linked to neutrophilic airway inflammation and lower lung function			(105)
	Thunderstorm asthma can be triggered by outdoor pollen, and possibly fungal, spores	(106, 107)	(106, 107)	(106, 107)
Food allergens (*n* = 170), e.g.,	In asthma, c/w non-atopy, odds for current asthma increased 3.8-fold if food allergy was likely	(108)	(108)	(108)
- Egg white -Peanut	Food allergy is an uncommon trigger in asthma, but may present as life-threatening asthma, especially to peanut and other tree nut allergens	(109, 110)		
- Tree nuts - Shellfish - Cows milk	Co-existing, poorly-controlled asthma is a risk factor for severe or fatal food-induced anaphylaxis	(110, 111)		
Occupational sensitizing agents (with latency)	Extensive lists of occupational asthmagens known to cause new-onset occupational asthma and/or exacerbate pre-existing asthma^†^ - Sensitizing HMW agents (e.g., plant allergens like flour, flowers, latex; animal allergens by animal handlers and lab workers; biological enzymes; fungi-yeasts) - Sensitizing LMW agents (e.g., chemicals like isocyanates, reactive dyes, industrial cleaning/sterilizing agents; metals; pharmaceuticals like antibiotics, opiates; solder flux; wood dusts)		(112–115)	(112–115)
**NON-ALLERGIC**				
Non-allergic triggers
- Respiratory viral infections - Cold air - Humidity - Exercise	Typically trigger variations in asthma symptoms and airflow limitation, and their presence increases the probability that the individual has asthma	(94)	(94)	(94)
Tobacco smoke exposure- Parental smoking	Parental smoking linked to increased incidence of childhood asthma and wheeze	(116)		
- Second-hand smoke exposure	Incident asthma from regular smoking from late childhood; may be greater for those non-allergic compared with allergic, and for those exposed to maternal smoking *in utero*	(117, 118)	(117, 118)	
- Personal smoking	Personal smoking may worsen asthma control/ exacerbations		(119)	(119)
	Personal smoking predisposes to post-BD airflow obstruction and asthma-COPD overlap			(120)
Traffic-related air pollution (TrAP)	Air pollutants (O_3_, PM_2.5_) linked to new asthma cases and increased ED admissions globally	(121)	(121)	(121)
-Car exhaust fumes	Long-term exposure to PM_1_ may worsen asthma, especially for young males with childhood allergy	(122)	(122)	
	Pollution-related decline in FEV_1_ growth was similar between those with and without asthma	(123, 124)	(123, 124)	
	Residential TrAP may contribute to new-onset and persistence of asthma in middle-aged adults			(125)
	Natural experiment of diesel exhaust exposure and adverse short-term changes in spirometry			(126)
Household air pollution (HAP)	Non-polluting home heating improved asthma symptoms, days off school, and healthcare utilization	(127)		
Occupational agents (no latency)	Airway irritants (e.g., chlorine, ammonia) - Irritant occupational asthma - Work exacerbated (pre-existing) asthma		(113, 114)	(113, 114)

*BD, bronchodilator; COPD, chronic obstructive pulmonary disease; HMW, high molecular weight; FEV_1_, forced expiratory volume in 1 second; LMW, low molecular weight; O_3_, ozone; PM, particulate matter*.

† *A web-based list of asthmagens can be found at www.occupationalasthma.com*.

### Parental and Personal Smoking

*In utero* maternal smoking and parental smoking in early life has been shown to be temporally associated with increased asthma in young children (116). Recent evidence from multi-generational studies suggest that grandmaternal smoking while the mother is *in utero* and paternal smoking during his adolescence can independently increase the risk of subsequent offspring childhood asthma. These findings suggest that tobacco smoking may cause heritable modifications of the epigenome, which increase the risk of asthma in future generations (128).

Smoking also seems to interact with sex. Female smokers had a higher prevalence of asthma than female non-smokers, but this difference was less frequent for males, suggesting that females may be more susceptible. Many studies have found that personal smoking predisposes an individual to increased risk of incident or new-onset asthma, although smoking-onset in adolescence, or adulthood typically occurs after early-onset asthma (119). As non-atopic asthma becomes increasingly common compared with atopic asthma in adults, this is most likely because this phenotype frequently coincides with a substantial history of cigarette smoking and its potential to predispose to chronic airflow limitation (119, 120, 129). Smokers with asthma form a distinct group that are more likely to have suboptimal asthma control (119) and develop asthma-COPD overlap syndrome (ACOS) in later life, characterized by incompletely reversed airflow obstruction following an inhaled bronchodilator (130).

From an epidemiological viewpoint, smoking is common in people with asthma, with around one-quarter of adults from 70 countries receiving recent asthma treatment also reporting to be current smokers (2). Some evidence suggests that people with asthma may be more likely to smoke, and this was seen especially in adolescents who have more severe disease (130).

### Outdoor Air Pollutants

Outdoor air pollution almost certainly has a major global impact on asthma for children and adults, especially in China and India (121). Worldwide, in 2015, 9–23 million and 5–10 million annual asthma emergency room visits have been attributed to the outdoor air pollutants ozone and particulate matter with an aerodynamic diameter <2.5 μm (PM_2.5_), respectively.(Exposure to PM_1_ has been found to increase the risk of asthma and asthma-related symptoms, especially among boys, and those with allergic predisposition (122). Residential markers of traffic-related air pollution, including nitrogen dioxide (NO_2_) exposure and distance to major roads, have been associated with increased risk for new-onset asthma, persistence of asthma and current asthma in a middle-aged, asthma-enriched, population-based cohort (125). In a natural experiment of 60 young to middle-aged adults with mild-to-moderate asthma, when compared with walking in the less polluted Hyde Park in London, walking along Oxford Street was associated with reductions in lung function, neutrophilic inflammation and airway acidification (126). These changes were greater for individuals with moderate asthma compared with mild disease at baseline.

### Outdoor Allergens

Exposure to ambient grass pollen is an important trigger for childhood asthma exacerbations requiring emergency department attendance and this has been recently confirmed by a systemic review (99). There is also scant evidence on the role of early life exposure to pollen in the development of childhood asthma (131). However, less evidence is available on the role of pollen in adult asthma (132), except in “Thunderstorm asthma” which is related to a combination of factors as described below.

In relation to other outdoor allergens, increasing evidence indicates that asthmatic children are susceptible to exacerbations that lead to hospitalization when exposed to outdoor fungal spores (104). Furthermore, high concentrations of outdoor fungal/mold exposure on peak days have been linked to asthma exacerbation and mortality in adults (103, 133, 134). IgE sensitization to fungal species is associated with increased asthma severity, neutrophilic inflammation, and reduced lung function consistent with ACOS (105).

### Thunderstorm Asthma

Thunderstorm asthma is defined as epidemics that occur during or shortly after a thunderstorm, where individuals affected would experience asthma-related symptoms such as breathlessness, wheezing and coughing. “Thunderstorm asthma” (106, 107) is the outcome of a complex interaction between multiple factors but not necessarily any one of them individually. Under certain weather conditions such as a thunderstorm, pollen grains may swell and burst to form fine respirable particles that are sufficiently small to enter the lower respiratory tract and precipitate severe asthma in those susceptible. This can occur in sensitized individuals who may or may not have a prior history of asthma or asthma symptoms, but who often have a history of allergic rhinitis. Fungal spore allergens may also be involved (133, 134).

On the 21st of November 2016, Melbourne, Australia, experienced a thunderstorm asthma health emergency (106, 107) that exceeded all previously reported thunderstorm asthma events [mainly in the UK and Australia (135, 136)]. In addition to a 4.3-fold increase in emergency attendances for acute respiratory distress symptoms after adjustment for temporal trends (107), nine deaths over the subsequent 10-day period were attributed to asthma as the primary cause (137). This mortality statistic was 50% more than expected based on the average for the same period over the previous 3 years (137), with a total of 10 deaths (immediate and delayed) attributed to the specific epidemic.

### Indoor Environment

Indoor pollutants such as products of combustion, including PM and NO_2_, and airborne allergens have been the subject of intense scrutiny as determinants of asthma given that most of our time is spent indoors.

There is substantial evidence to suggest that indoor allergens generated by house dust mite, mold and cat are triggers for both childhood and adult asthma, especially in those sensitized (100–102). However, their role in the etiology of asthma is not clear. On the other hand, primary prevention trials on reduction of allergen exposure in early life have failed to detect any benefits. Some observational studies have even reported exposure to allergens in infancy may help develop tolerance and reduce the risk of asthma. However, the evidence is not consistent. Interestingly, there is increasing evidence on this tolerance hypothesis in the etiology of food allergy in which a clinical trial has shown that early consumption of peanuts can reduce the development of peanut allergy (138, 139). These findings suggest that it may be worth exploring this notion of early exposure to allergens leading to development of tolerance, which in turn may reduce the risk of developing asthma.

### Occupational Exposures

Occupational exposures to asthmagens or inciting sensitizing agents are common and often under-recognized causes of work-related asthma (WRA). WRA includes two distinct subtypes: work-aggravated/exacerbated asthma (WEA) occurring in individuals with pre-existing asthma, and occupational asthma (OA) occurring in individuals without previous asthma. OA is typically subclassified into immunoglobulin (Ig)-E-mediated or sensitizer-induced OA (90%) and irritant-induced occupational asthma (10%) (140). A diagnosis of WRA requires the objective diagnosis of asthma with symptoms temporally related to the individual's place of employment (141). Over 250 agents may potentially cause sensitization and possibly occupational asthma (OA), and comprehensive lists are available (Table 1) (112–115). Briefly, the two main classes of sensitizing agents, namely high molecular weight (HMW) and low molecular weight (LMW) agents can cause sensitizer-induced asthma which is usually after a latency period and this may contrast the frequent rapid action of irritant agent exposure. A web-based list of agents can be found at www.occupationalasthma.com.

Differentiating sensitizer-induced OA from WEA can be a major challenge for managing clinicians. The time-to-diagnosis of sensitizer-induced OA varies but is usually made between 2 and 4 years following the onset of work-related symptoms, and this timeframe is substantially shorter for the diagnosis of WEA as these individuals are usually medically managed for pre-existing asthma (142). Among compensation claims, confirmed OA diagnoses most have a causative sensitizing agent identified (143).

Despite challenges in estimating the true incidence of OA, around 10–20% of all adult-onset asthma is thought to be caused by respiratory sensitizers and/or irritants in the occupational setting. Of note, this figure can vary widely (from 4 to 58%) (144) and is largely derived from populations in high income countries (144–146). To contrast, work-related exacerbations can occur frequently in 20–25% of working adults who have pre-existing asthma (147), although objective evidence of poorer asthma control is often difficult to demonstrate (140). While past under-recognition and/or under-reporting of OA might have obscured changing trends over recent decades, the health care industry has successfully reduced the risk of latex-induced allergy and OA by substituting natural rubber latex (NRL) gloves for powder-free, protein-poor NRL gloves. This successful approach for exposure minimization highlights the benefit of identifying those at risk from occupationally-related asthma and minimizing potentially harmful exposures.

### Lifestyle Factors

Although already mentioned as an “asthma-plus” co-morbidity, the prevalence of obesity in countries in which a Westernized diet predominates is now of epidemic proportions. These dietary patterns feature a high calorie intake which is high in saturated fat and refined sugars and associated with a high glycaemic index, as well as low nutritional value in terms of dietary fiber and vitamins. While this “obesogenic diet” may lack antioxidant and anti-inflammatory properties (148), a meta-analysis has found being overweight and obese to be associated with a dose-response increase in incident asthma in adults (149). While this review did not find significant sex-related differences, female obesity has been associated with a pauci-eosinophil and non-atopic asthma endotype that is symptom-predominant and less steroid-responsive in previous cluster and LCA (16, 28). For all individuals with otherwise poorly controlled asthma, the behavior of avoiding strenuous exercise might confuse severe disease with well-controlled asthma, and this in turn can lead to poorer fitness levels and a propensity to weight gain (94). This is of particular importance to children with asthma, at a time when lifestyle patterns are being especially shaped by external factors.

The role of infant breastfeeding in the prevention of asthma is debated, however this has been largely clarified by findings from the TAHS cohort. This longitudinal study of participants who were followed between childhood and middle-age showed that breast feeding reduced the risk of childhood asthma and conversely increased the risk of adult asthma, but for only those with a familial predisposition (150). In 2015, a systematic review summarized the overall estimate for a longer compared with shorter duration of breastfeeding to be modestly protective for asthma in later childhood-adolescence [odds ratio 0.90 (95%CI 0.84–0.97), *I*^2^ = 63%] (151). While the effect was stronger when restricted to studies from lower-to-middle income countries, no association was seen when restricting the meta-analysis to only cohort studies. The overall conclusion was that the evidence was of low quality. The authors primarily hypothesized that breastfeeding-related reductions in childhood wheeze might relate to the known beneficial immunological factors which could reduce childhood viral infections that predispose to asthma.

## Impact of Childhood and Adult Asthma on Lung Function Trajectories and COPD

### Childhood Asthma and Lung Function

Studies that have investigated the impact of childhood asthma on lung function from childhood to adolescence have found that different asthma phenotypes differentially impact long-term lung function outcomes. This is particularly relevant to longitudinal asthma phenotypes, which earlier studies attempted to identify by manually classifying the change of symptoms, but more recent studies have identified distinct longitudinal phenotypes using advanced statistical techniques such as Latent Class Analysis (LCA) as mentioned above. Overall, the use of LCA has led to the identification of more asthma phenotypes and therefore has helped to better disentangle the long-term effects of childhood asthma. The majority of studies have shown that persistent wheeze is related to reduced lung function development throughout adolescence (31, 45, 152, 153), while some suggest the effects of persistent wheeze and relapsed wheeze on lung function are established from mid-childhood, without further decline in tracking of FEV_1_ over time (154, 155). It has also been reported that childhood asthma associated with allergic comorbidities, such as eczema and allergic rhinitis, has persistent lung function impairment from birth to adolescence as compared to asthma without such comorbidities (156). These findings have led to the hypothesis that asthma with atopic dermatitis and allergic rhinitis may represent a specific phenotype originating *in utero* (156).

Several longitudinal studies have investigated the long-term impacts of childhood asthma on lung function decline and COPD. Childhood asthma has been associated with adult lung function deficits and increased risk of COPD (157–159). While a number of studies have reported that childhood asthma itself has no impact on adult lung function decline (154, 157, 158, 160), a study that collected childhood asthma status retrospectively has reported that it is associated with greater lung function decline, which may be related to recall bias (161). More recent findings suggest that childhood asthma is related to longitudinal lung function trajectories that are “below normal” within both the general population (162) and asthmatics (163).

Overall, current evidence suggests that many children with childhood asthma/wheeze, especially early persistent asthma/wheeze, may have reduced airway and lung development and not reach their peak lung function potential as influenced by pre-determined lung function trajectories. These lung function deficits may track (or persist) into adulthood without additional progressive loss. However, it is not clear whether childhood asthma can directly affect the rate of lung function decline unless it continues as adult asthma.

### Adult Asthma and Lung Function

While asthma in adults is often the persistence or relapse of asthma from childhood, “true” adult onset asthma is a distinct phenotype most often related to environmental risk factors such as smoking (164). The impact of adult asthma on lung function outcomes appears to vary by phenotype including age-at-onset. It has been shown that both early and late onset adult current asthma were associated with a reduction in lung function and an increased risk of fixed airflow obstruction at 45 years, with the effect of early onset asthma being greater than late onset asthma (165–167). These findings differ from the above mentioned systematic review and meta-analysis which found greater levels of fixed airflow obstruction for those with late-onset adult asthma, which most likely relates to inaccurate retrospective recall of childhood asthma by adults (164).

Further evidence from longitudinal studies suggests that adults with asthma have greater lung function decline than those without asthma (168–170). While both early and late onset adult asthma seem to be associated with faster lung function decline, the decline associated with early onset adult asthma is greater than that with late onset adult asthma (167).

An important question is whether we can disentangle the components of lung function deficits in adults with asthma over the life course. Lung function deficits in those with early onset adult asthma may result from both the tracking of reduced lung function from childhood and additional loss from a greater rate of decline in adulthood (167, 171). It has been suggested that adults who developed new-onset asthma had reduced lung function at baseline (172, 173), but it is unknown whether lower lung function before early adulthood, in the absence of childhood asthma, predisposes to “true” adult onset asthma. However, asthma/wheeze status in early life is often forgotten by adults leading to misclassification of “true” adult-onset asthma (20). On the other hand, lung function deficits in adult-onset asthma after peak lung function has been attained are also likely to be due to faster lung function decline.

## Other Health Impacts of Childhood and Adult Asthma

Another major impact of asthma is through its associated additional morbidities, including a predisposition to serious infections such as bacterial pneumonia from a higher nasopharyngeal carriage of *Streptococcus pneumoniae* (174). Although not well-understood, asthma-related chronic airway inflammation with damaged airway mucosa and immunomodulating treatments such as inhaled corticosteroids have been implicated, and lower antibody levels in response to the Pneumococcal vaccine have also been observed (174). In addition to a 2.4-fold increased risk for invasive pneumococcal disease (175), susceptibility to respiratory and non-respiratory infections (such as Herpes zoster and *E. coli* bacteraemia) in never smokers with asthma has been compared with the relative risk of diabetes (176). This susceptibility to infection supports the hypothesis of weaker T_H_1 immune responses associated with T_H_2-related disease. However, recommendations for Pneumococcal immunization are inconsistent (94) suggesting more evidence is needed to gain consensus of its benefits (177, 178).

Adult asthma is also associated with a number of chronic conditions. The associations between childhood asthma and allergies such as eczema and food allergy are very well-established. Adult asthma is known to be commonly associated with diabetes, osteoporosis, metabolic syndrome, cardiovascular diseases, and issues with mental illness such as anxiety and depression while there are a number of other morbidities that have been linked (179). When a chronic condition is present in people with asthma, that is known as asthma co-morbidity. Almost two-thirds (62.6%) of patients with asthma have at least one comorbid condition with 16% having four co-morbidities (180). This prevalence of these morbidities in asthmatics is too high to be simply due to the chance development of chronic conditions while aging but these associations do not imply causality. The etiology of asthma co-morbidities may be linked to asthma itself, other morbidities, shared mechanisms, shared environmental, and/or shared genetic risk factors. Regardless of the etiology, it is well-known that asthma comorbidities are associated with worse outcomes for the patients and the healthcare systems (181), and managing asthma comorbidities has been associated with significant improvement in its prognosis. Revising guidelines on to handle comorbidities may lead to a more targeted treatment for comorbidities and more patient-centered asthma management, which in turn lead to better outcomes.

## Summary

The evidence on the trends and environmental determinants for childhood and adult asthma are similar, although the evidence is stronger for childhood asthma, which is partly related to the stronger attention that childhood asthma has received from the research community. The global epidemic of asthma is continuing, especially in low to middle income countries, although it has subsided in some high income countries. Epidemiological research has helped to uncover some important environmental determinants that trigger asthma, but the role of environmental factors in the etiology of asthma remains largely unknown. Research into interactions between potential determinants may help tease out the etiology. Therefore, there is an urgent need to further investigate the complex mechanisms driving the interrelationship between environmental and genetic determinants to identify high risk groups and key modifiable exposures. Given the long term impact of both childhood and adult asthma, we would argue that our focus going forward to reduce the health burden of asthma should be firmly on improving not only short-term symptoms, but also the long-term respiratory and other health outcomes (182).

## Author Contributions

The authors alone are responsible for the content and writing of the article. AC, SD, and JP wrote sections of the initial draft of the manuscript which was then critically revised for flow and important content areas by SD and JP. All authors approved the final version of the manuscript.

### Conflict of Interest Statement

AC has received personal fees for consultancy from Regeneron/Sanofi, Philips and Boehringer Ingelheim; consultancy and speaker fees from Novartis; and speaker fees from Thermo Fisher Scientific. JP has received a travel grant from Boehringer Ingelheim. The remaining author declares that the research was conducted in the absence of any commercial or financial relationships that could be construed as a potential conflict of interest
